# Supplementary material for: The causal relationship between the human gut microbiota and pyogenic arthritis: a Mendelian randomization study
Source: Front Cell Infect Microbiol. 2024 Nov 26;14:1452480. doi: 10.3389/fcimb.2024.1452480 (PMC11629706; doi:10.3389/fcimb.2024.1452480)
Supplement: Supplementary file 1 [file DataSheet1.zip › mendelian test/ebi-a-GCST90027558.csv_leave_one_out.pdf]

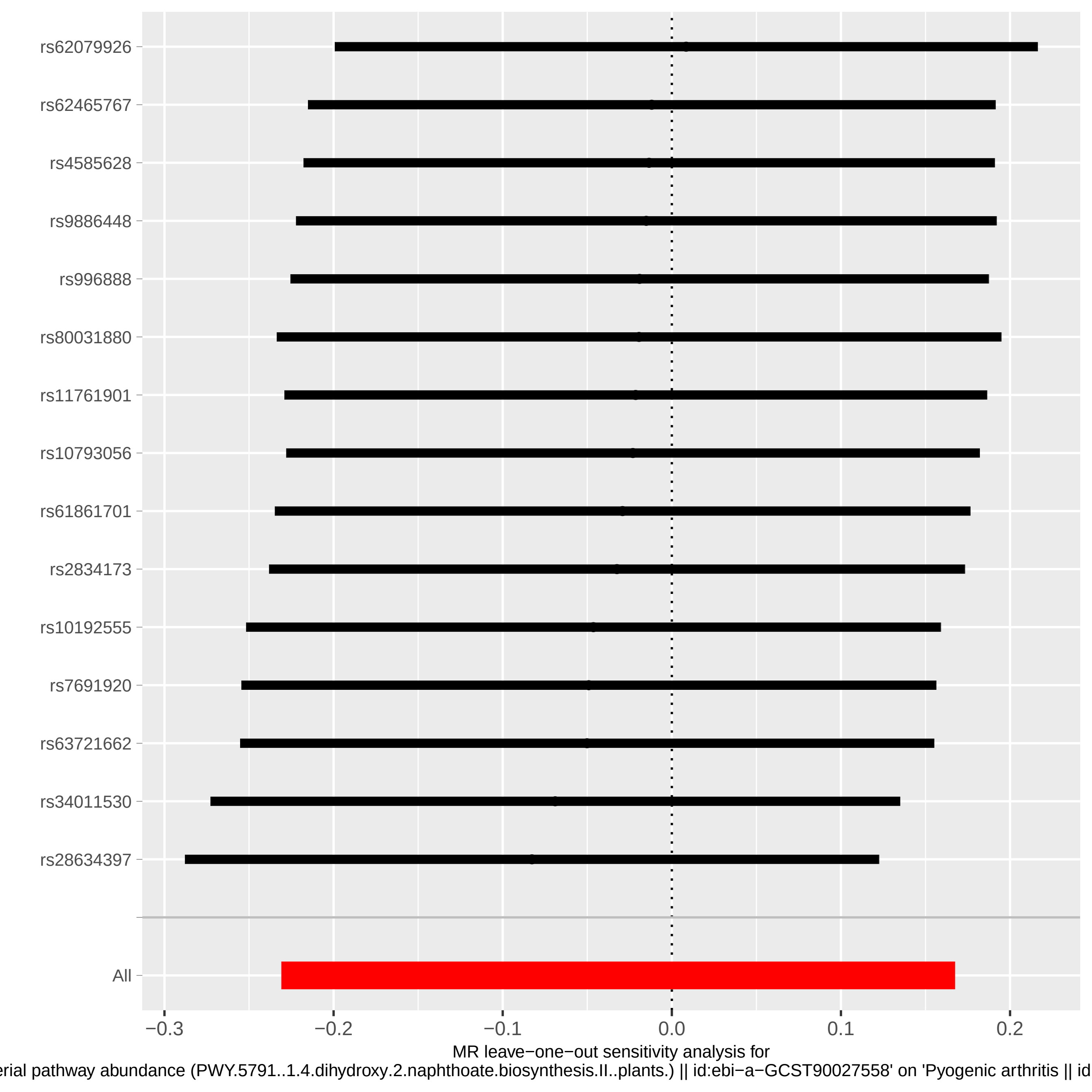
rs62079926
rs62465767
rs4585628
rs9886448
rs996888
rs80031880
rs11761901
rs10793056
rs61861701
rs2834173
rs10192555
rs7691920
rs63721662
rs34011530
rs28634397
All
−0.3
−0.2
−0.1
0.0
0.1
0.2
MR leave−one−out sensitivity analysis for
erial pathway abundance (PWY.5791..1.4.dihydroxy.2.naphthoate.biosynthesis.II..plants.) || id:ebi−a−GCST90027558' on 'Pyogenic arthritis || id
